# A Tribute to Dr. George Cupples

**Published:** 1918-10

**Authors:** B. F. Kingsley

**Affiliations:** San Antonio, Texas


### A Tribute to Dr. George Cupples.

B. F. KINGSLEY, M. D., SAN ANTONIO, TEXAS.

On a former occasion some fourteen years ago was held a memorial service for Dr. George Cupples, in his life one of the greatest physicians, surgeons and citizens in the great State of Texas.

From a sense of deep personal loss I dared not trust myself to lay my tribute of love and affection on the altar of his memory at that time, thinking also that another opportunity would offer.

For sixteen long years scarcely a day passed that I did not assist Dr. Cupples in some operation or meet him in consultation or that he did not so assist or consult with me. This long and intimate association should fit me to speak of and picture the salient, the beautiful and the priceless features of this illustrious character. Yet no one realizes more than I how inadequate are any words I may utter to do justice to this prince of physicians and of men!

It was my good fortune to first meet Dr. Cupples early in February, 1877, soon after which I was examined by him as President of the first Board of Medical Examiners for this district. He was largely instrumental in having passed a law creating district boards; I believe the first law governing the practice of medicine in Texas.

I shall never forget his kindly feeling and interest in me, inviting me to call upon him, and to assist him and with what keen interest he entered into the discussion of medical subjects, and how he surprised me and others in our constant relations with him in his profound and extensive technical knowledge in all the branches of medical science, and his deep and comprehensive knowledge of literary and scientific questions in general.

Having offices at the same place and part of the time occupying the same office our association was almost constant over this period. It was when I first met him that he was foremost in the efforts to organize the Western Texas Medical Association—the result of which was an application for a charter dated December 29th, 1876, and signed Dr. Geo. Cupples, Dr. T. R. Chew, Dr. J. J. Gaenslen, Dr. J. Jordan, Dr. Ferd Herff, Dr. R. C. Campbell, Dr. Edward Bennett—which charter was approved Feb. 28th, 1877. A few months thereafter it was my good fortune to join this association and still further my good fortune to follow its history and its vicissitudes through its twenty-five years of life. If I am not mistaken he was its first President. Throughout its history he was the most faithful and persistent in his attendance, the most frequent and able contributor of papers, the most complete, thorough and convincing in his discussions, suave, always able and ready, always genteel, and punctilious, in all his social, professional and ethical relations. He invariably preserved his equilibrium of bearing and manner in every relation and circumstance.

His early classical and military training gave him a breadth and scope of information possessed by few men. Eleven years prior to the foundation of the Western Texas Medical Association he was an active factor in the organization of the Texas State Medical Association and one of its earliest presidents, in fact he was twice President. He was constantly chairman of some section, or important committee, and his advice was sought on all occasions. In fact I think no other man wielded such a profound and far reaching influence for good as Dr. Cupples. He worked hard for years in and through medical associations and outside among his many friends to obtain legislation affecting sanitary and medical matters generally, and has left a deep and indelible impression upon the profession and its evolution in this State.

He was progressive and untiring in his study and effort to keep abreast with medical science, and as an instance compiled in his later years the surgical history of Texas, in the preparation of which it took him months of weary study far into the night. Drs. Spring and Berry can testify with me as to the great value and laborious character of that work. His careful and painstaking methods and technique in surgery are illustrated by his splendid results, and that in over fifty years of extensive surgical practice he never lost a case from anesthesia and he seldom used anything but chloroform.

He was one of the first, if not the first to use sterilized water to wash out the adbodmen and leave some in the cavity after laparotomy for septic conditions.

He had a will of adamant, and a constitution of steel; he never tired in an operation, or complained of fatigue from overwork. The picture of this frail and aged man ascending a long flight of stairs, two steps at a time with the agility of youth, always remain fresh in my memory. One of the last operations he did was a combined perineal section and laparotomy for urinary extravasation resulting from a supra-pubic puncture of the bladder for retention of urine from stricture, in the midst of the most unsanitary surroundings, in which large quantities of water were used to flush out the abdominal cavity, and yet with the most brilliant results.

He never lost an opportunity to defend the honor, and to uphold the dignity of the profession. In all particulars he was as great and fine a citizen and gentleman as he was a surgeon. He always insisted that the doctor and surgeon should be paid in a measure commensurate with other professions, and yet he did a vast amount of charitable work. Through his great skill and experience, combined with that of that other great surgeon, Dr. Ferdinand Herff, the reputation of San Antonio as a surgical centre has been known throughout the State, the country and throughout Mexico for sixty years, and has attracted hither thousands who come for medical and surgical relief.

Dr. Cupples' character was exemplified by three cardinal virtues—dignity, charity and justice.

He never went back on a friend, he was liberal to a fault, and his hospitality knew no bounds. There was absolutely nothing small in his nature. In the limited time at my command, many beautiful traits of character and virtues must remain unspoken, but from my heart and an intimate knowledge of him, I must exclaim with that former distinguished citizen of San Antonio, General Young, upon whom it was my privilege to assist Dr. Cupples in operating, and whose recovery was prompt and perfect: "That it is a great pleasure for me to pay my tribute to the man I esteem really and truly the greatest physician that Texas has ever known."

The honors that have been conferred on the great inventors, discoverers and thinkers in medicine have through all ages been grudgingly given, yet medicine has given to the world many of its greatest benefactors, and I think the time has come and the opportunity is ripe for this profession to initiate a movement looking towards the erection of a monument in some public place in this city to commemorate his noble virtues and example, as physician and citizen. No man was more popular in the profession. In every act and thought he always had the best interests of the community and the profession at heart.

